# Genome-wide generation and genotyping of informative SNPs to scan molecular signatures for seed yield in chickpea

**DOI:** 10.1038/s41598-018-29926-1

**Published:** 2018-09-05

**Authors:** Udita Basu, Rishi Srivastava, Deepak Bajaj, Virevol Thakro, Anurag Daware, Naveen Malik, Hari D. Upadhyaya, Swarup K. Parida

**Affiliations:** 1 National Institute of Plant Genome Research (NIPGR), Aruna Asaf Ali Marg, New Delhi, 110067 India; 2 International Crops Research Institute for the Semi-Arid Tropics (ICRISAT), Patancheru, 502324 Telangana India

## Abstract

We discovered 2150 *desi* and 2199 *kabuli* accessions-derived SNPs by cultivar-wise individual assembling of sequence-reads generated through genotyping-by-sequencing of 92 chickpea accessions. Subsequent large-scale validation and genotyping of these SNPs discovered 619 *desi* accessions-derived (DAD) SNPs, 531 *kabuli* accessions-derived (KAD) SNPs, 884 multiple accessions-derived (MAD) SNPs and 1083 two accessions (*desi* ICC 4958 and *kabuli* CDC Frontier)-derived (TAD) SNPs that were mapped on eight chromosomes. These informative SNPs were annotated in coding/non-coding regulatory sequence components of genes. The MAD-SNPs were efficient to detect high intra-specific polymorphic potential and wide natural allelic diversity level including high-resolution admixed-population genetic structure and precise phylogenetic relationship among 291 *desi* and *kabuli* accessions. This signifies their effectiveness in introgression breeding and varietal improvement studies targeting useful agronomic traits of chickpea. Six trait-associated genes with SNPs including quantitative trait nucleotides (QTNs) in combination explained 27.5% phenotypic variation for seed yield per plant (SYP). A pentatricopeptide repeat (PPR) gene with a synonymous-coding SNP/QTN significantly associated with SYP trait was found most-promising in chickpea. The essential information delineated can be of immense utility in genomics-assisted breeding applications to develop high-yielding chickpea cultivars.

## Introduction

Chickpea (*Cicer arietinum*) is a self-pollinated, diploid (2n = 16) and economically important legume food crop rich in human dietary proteins^1^. The chickpea is primarily represented by its *desi* and *kabuli* cultivars exhibiting a distinct differentiation in both agro-morphological and architecture traits, which genomes have been sequenced recently^2–5^. Substantial enhancement of its yield and productivity by developing high-yielding a/biotic stress tolerant and climate-ready cultivars is essential at present to sustain the global food security. To accomplish these objectives, many significant efforts involving classical genetics as well as genomics-assisted breeding strategies like quantitative trait loci (QTL) mapping, fine-mapping/map-based cloning and association analysis have been made to decipher the inheritance pattern and quantitative dissection of complex yield and stress tolerance traits for genetic improvement of chickpea^6–30^. However, these are constrained by narrow genetic base and low intra-specific marker polymorphism among *desi* and *kabuli* accessions due to combined strong impact of four major domestication bottlenecks in chickpea as compared to other food crop plants^2,3,31–35^. To overcome the aforesaid limitations, development and high-throughput genotyping of large-scale sequence-based informative markers like single nucleotide polymorphism (SNPs) exhibiting a higher potential of polymorphism among cultivated *desi* and *kabuli* accessions is a prerequisite for their use in genomics-assisted breeding applications and genetic improvement of chickpea.

**Figure 1:**
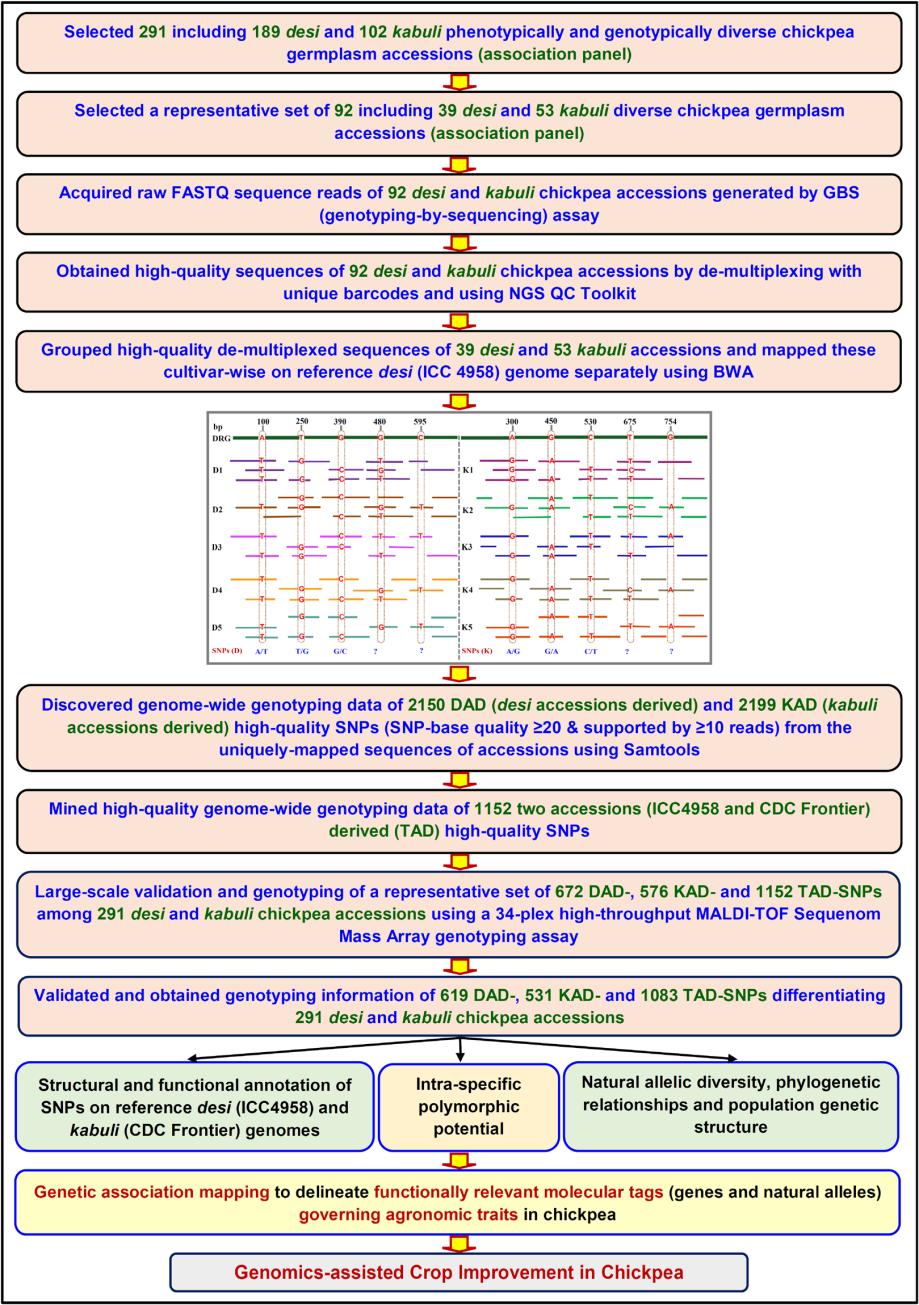
Strategy adopted in the present study to generate high-quality genotyping data of diverse kinds of informative SNPs (DAD-, KAD-, MAD- and TAD-SNPs) from 291 *desi* and *kabuli* accessions at a genome-wide scale to be deployed for multiple genomics-assisted breeding applications in chickpea.

The next-generation sequencing (NGS)-led genome and transcriptome sequencing of multiple cultivated (*desi* and *kabuli*) and wild accessions are found efficient in fast discovery of numerous SNPs at a genome-wide scale in chickpea^2,3,13,18,30,36–44^. The large-scale validation and high-throughput genotyping of these SNPs mined from limited number of sequenced accessions using diverse array-based genotyping assays commonly exhibit less potential of inter-/intra-specific polymorphism and natural allelic diversity among cultivated and wild accessions to be deployed efficiently for genetic enhancement of chickpea^7,10–14,17–23,25–27^. However, degree of these polymorphic and diversity potential can be enriched by using the genotyping information of SNPs derived from whole genome resequencing data that are generated from a large number of *desi*, *kabuli* and wild accessions of chickpea. Essentially, this approach requires huge cost, time and resources for developing and genotyping of SNPs at a whole genome/gene level in chickpea. In this regard, NGS-based low sequencing-depth genome coverage sequencing strategies like genotyping-by-sequencing (GBS) assay seems quite promising for rapid discovery and genotyping of genome-wide SNPs simultaneously among numerous cultivated (*desi* and *kabuli*) and wild accessions in order to drive high-throughput genetic analysis in chickpea with sub-optimal expense of cost, labour and resources^7,10–14,17–23,25–27^. However, these low genome coverage sequencing assays often generate erroneous and non-uniform SNP genotyping data across accessions, thereby further large-scale revalidation and genotyping of GBS-derived SNPs is needed currently prior to their use in genetic improvement of chickpea. In this perspective, high-throughput validation and genotyping of numerous GBS-derived high-quality SNPs discovered by assembling all the sequence reads generated from large number of accessions belonging to each of the *desi* and *kabuli* cultivars individually (cultivar-wise) can be useful for their deployment in genomics-assisted crop improvement of chickpea. In view of aforementioned prospects, efforts were made in the current study for large-scale validation and high-throughput genotyping of informative SNPs, discovered by *desi* and *kabuli* cultivar-wise individual assembling of the sequence reads, generated from 92 accessions using GBS assay at a genome-wide scale in chickpea (Fig. 1). The efficacy of these validated whole genome/gene-derived SNPs to detect intra-specific polymorphism, molecular diversity and domestication pattern among 291 *desi* and *kabuli* cultivated chickpea accessions and for establishing marker-trait association with seed yield per plant were determined.

## Results

### Genomic distribution of informative SNPs in chickpea

The sequence reads generated by sequencing and genotyping of 92 *desi* and *kabuli* chickpea accessions using GBS assay were assembled cultivar-wise individually. This exertion successfully discovered various types of informative SNPs including *desi* accessions-derived (DAD)-, *kabuli* accessions-derived (KAD)- and multiple accessions-derived (MAD)-SNPs which were further compared with two accessions (ICC 4958 and CDC Frontier) derived (TAD) SNPs for evaluating the potential of these developed markers in large-scale genetic analysis in chickpea (Fig. 1).

To develop these informative markers primarily, the sequencing data generated from 92 including 39 *desi* and 53 *kabuli* chickpea accessions using a GBS assay were de-multiplexed and quality filtered. As a result, 207.9 million processed high-quality sequence reads uniformly distributed across 92 chickpea accessions were obtained which altogether covered 28-fold (X) sequence depth of coverage. This exertion overall produced 3.3 to 5.7 million of high-quality sequence reads per accession covering on an average 5.4-fold (X) sequence depth of coverage. More than 85% of de-multiplexed high-quality sequence reads generated from accessions were mapped separately (*desi* and *kabuli* cultivar-wise) to unique physical locations on *desi* reference genome^4^. The *desi* and *kabuli* cultivar-wise reference genome-based GBS analysis altogether detected 2150 (DAD-SNPs) and 2199 (KAD-SNPs) high-quality SNPs (supported by read-depth ≥10, SNP base quality ≥20, <1% missing data and ~2% heterozygosity in each accession) from the accessions which were further validated through PCR amplicons-based sequencing assay. For large-scale validation and high-throughput genotyping, a representative set of high-quality 672 DAD-, 576 KAD- and 1152 TAD-SNPs differentiating 39 *desi*, 53 *kabuli* and 2 (ICC 4958 and CDC Frontier) accessions, respectively, were selected based on their uniform physical distribution on eight chromosomes. These physically mapped SNPs were genotyped using the genomic DNA of 291 *desi* and *kabuli* chickpea accessions through a 34-plex high-throughput matrix assisted laser desorption ionization-time of flight (MALDI-TOF) Sequenom Mass Array genotyping assay. The optimized multiplex assay successfully validated 619 DAD-, 531 KAD- and 1083 TAD-SNPs based on their homozygous and heterozygous allele discrimination as evident from the call cluster plots of low and high mass homo- and hetero-zygote yields with a 92–94% genotyping success rate (Tables 1 and S1–S4). The comparison of both DAD- and KAD-SNPs identified 884 MAD-SNPs differentiating all 92 *desi* and *kabuli* accessions together which were physically mapped on eight chickpea chromosomes (Tables 1 and S3). Therefore, all the DAD- and KAD-SNPs selected in this study were common to MAD-SNPs. However, only 266 SNPs were common between DAD- and KAD-SNPs while the remaining 353 DAD- and 265 KAD-SNPs were found to be unique. We could not obtain any DAD-, KAD and MAD-SNPs that are common to TAD-SNPs.

**Table 1:** Genomic distribution of SNPs physically mapped on eight chickpea chromosomes.

Chromosomes	Number (%) of SNPs mapped			
	*Desi* accessions (189)-derived (DAD) SNPs	*Kabuli* accessions (102)-derived (KAD) SNPs	Multiple accessions (291 *desi* and *kabuli*)-derived (MAD) SNPs	Two (*desi* ICC 4958 and *kabuli* CDC Frontier) accessions-derived (TAD) SNPs
*Ca_Chr01*	105 (17.0)	79 (14.9)	133 (15.0)	170 (15.7)
*Ca_Chr02*	79 (12.8)	64 (12.1)	109 (12.3)	108 (10.0)
*Ca_Chr03*	45 (7.3)	77 (14.5)	98 (11.1)	112 (10.3)
*Ca_Chr04*	183 (29.6)	148 (27.9)	260 (29.4)	474 (43.8)
*Ca_Chr05*	33 (5.3)	45 (8.5)	55 (6.2)	28 (2.6)
*Ca_Chr06*	72 (11.6)	45 (8.5)	97 (11.0)	49 (4.5)
*Ca_Chr07*	77 (12.4)	54 (10.2)	95 (10.7)	113 (10.4)
*Ca_Chr08*	25 (4.0)	19 (3.6)	37 (4.2)	29 (2.7)
**Total**	**619**	**531**	**884**	**1083**

*Ca_Chr*: *Cicer arietinum* Chromosomes.

**Figure 2:**
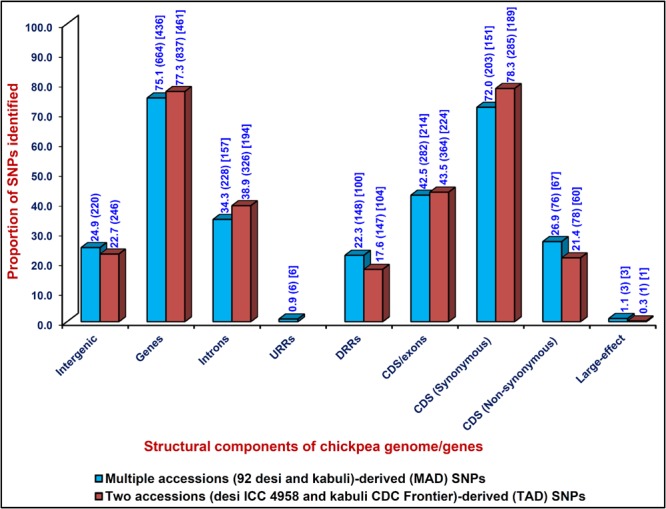
Proportionate distribution of MAD- and TAD-SNPs in different coding (synonymous and non-synonymous) and non-coding (intron, URR and DRR) sequence components of genes and intergenic regions annotated from *desi* chickpea genome. The gene annotation of *desi* genome^4^ was considered as reference to infer the coding DNA sequence (CDS)/exons, introns, up/downstream regulatory region (URR/DRR) sequence components of genes. Digits within the round and square Parentheses indicate the (number of gene-derived SNPs) and [number of genes with SNPs], respectively, representing each class of coding and non-coding regions of chickpea genes.

The detailed structural annotation of 884 MAD- and 1083 TAD-SNPs exhibited the occurrence of 664 (75.1%) and 837 (77.3%) SNPs in 436 and 461 chickpea genes, respectively (Fig. 2 and Tables S1–S4). The remaining 220 (24.9%) MAD- and 246 (22.7%) TAD-SNPs were present in the intergenic regions of chickpea genome. Both MAD- and TAD-SNPs (282–364 SNPs, 42.5–43.5%) especially discovered from the exons/CDS of chickpea genes (214–224) were found most abundant (Fig. 2 and Tables S1–S4). Among the coding MAD- and TAD-SNPs, a higher proportion (72–78.3%) of SNPs (203–285) in the genes (151–189) showed synonymous substitutions while remaining (21.7–28.0%) SNPs (76–78) in the genes (61–70) revealed both missense and large-effect nonsense substitutions (Fig. 2 and Tables S1–S4). The functional annotation of 436 and 461 genes with 664 MAD- and 837 TAD-SNPs, respectively, exhibited their highest correspondence to growth, development and metabolism-related proteins (61%) followed by transcription factors (20%) and signal transduction proteins (11%). Moreover, GO enrichment analysis of these genes with MAD- and TAD-SNPs revealed a significant overrepresentation/enrichment of GO terms in the genes associated with molecular function (nucleic acid binding, 27%, P: 1.2 × 10^−40^) followed by biological process (metabolism, 11.5%, 1.6 × 10^−50^) and cellular component (macromolecular complex, 5.7%, 1.0 × 10^−43^). The transcription factor (TF) genes with MAD- and TAD-SNPs belonging to TF families like basic helix-loop-helix (*bHLH*), basic leucine zipper (*bZIP*) and APETALA2 ethylene-responsive element binding proteins (*AP2-EREBP*) were found predominant.

### Natural allelic diversity and population genetic structure among *desi* and *kabuli* chickpea accessions

A set of 619 DAD-, 531 KAD-, 884 MAD- and 1083 TAD-SNPs were analysed to evaluate their potential of detecting polymorphism among 291 *desi* and *kabuli* chickpea accessions (Table 2). The 884 MAD-SNPs detected a higher degree of polymorphism as compared to that of DAD-, KAD- and TAD-SNPs among accessions which was clearly evident from their maximum level of PIC (varied from 0.10 to 0.48, mean 0.45) and high nucleotide diversity (mean θπ: 1.55) estimation (Table 2). Notably, 526 DAD-, 449 KAD-, 793 MAD- and 758 TAD-SNPs revealed high intra-specific polymorphic potential among 189 *desi* accessions (70–89.7% polymorphism, mean PIC: 0.30–0.47 and mean θπ: 1.29–1.59) than that of 102 *kabuli* accessions (64.3–73.3%, 0.27–0.41 and 1.23–1.48). Notably, a higher potential of MAD-SNPs followed by DAD-SNPs and a lower potential of TAD-SNPs for detecting polymorphism among accessions belonging to *desi* and *kabuli* cultivars was observed (Table 2).

**Table 2:** Polymorphism and molecular diversity potential of genome-wide informative SNPs estimated in 291 chickpea accessions using diverse statistical measures.

Accessions	DAD-SNPs				KAD-SNPs				MAD-SNPs				TAD-SNPs			
	Percent polymorphism	PIC	θπ	Genetic distance	Percent polymorphism	PIC	θπ	Genetic distance	Percent polymorphism	PIC	θπ	Genetic distance	Percent polymorphism	PIC	θπ	Genetic distance
All 291 accessions	619	0.10–0.41 (0.35)	1.36	0.13–0.64 (0.55)	531	0.10–0.38 (0.32)	1.32	0.15–0.62 (0.46)	884	0.10–0.48 (0.45)	1.55	0.12–0.79 (0.67)	1083	0.11–0.33 (0.29)	1.25	0.10–0.40 (0.37)
189 *desi* accessions	526 (85.0)	0.10–0.40 (0.36)	1.45	0.13–0.69 (0.58)	449 (84.5)	0.10–0.38 (0.32)	1.42	0.15–0.63 (0.52)	793 (89.7)	0.10–0.48 (0.47)	1.59	0.12–0.78 (0.69)	758 (70.0)	0.11–0.32 (0.30)	1.29	0.11–0.41 (0.39)
102 *kabuli* accessions	376 (60.7)	0.10–0.37 (0.33)	1.33	0.14–0.62 (0.49)	334 (62.9)	0.10–0.31 (0.30)	1.27	0.15–0.59 (0.44)	648 (73.3)	0.10–0.43 (0.41)	1.48	0.12–0.73 (0.58)	697 (64.3)	0.11–0.29 (0.27)	1.23	0.10–0.35 (0.31)

θπ: Average pair-wise nucleotide diversity.

PIC: Polymorphism information content.

**Figure 3:**
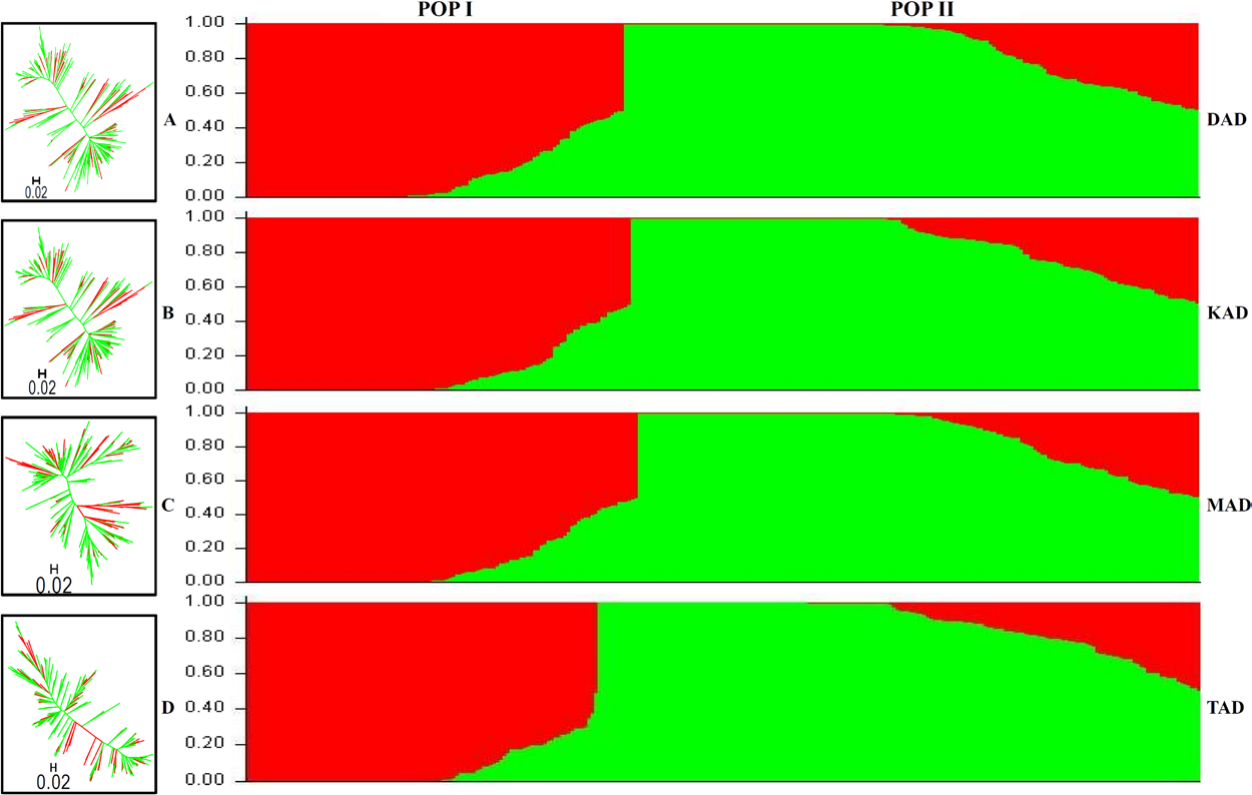
Unrooted phylogenetic tree and population structure (with population number K = 2) illustrating the evolutionary relationships and best possible population genetic structure, respectively, among 291 *desi* and *kabuli* chickpea accessions using 619 DAD (**A**) 531 KAD (**B**) 884 MAD (**C**) and 1083 TAD (**D**) SNPs. The mapped markers assigned accessions into two populations (POP I and POP II) which essentially corresponded to their geographical as well as cultivar-specific origination. Red and green colours correspond to POP I and POP II, respectively, as defined by both phylogenetic tree and population genetic structure. The accessions represented by vertical bars along the horizontal axis were classified into K colour segments as per their estimated membership fraction in each K cluster.

The molecular diversity and population genetic structure among 291 *desi* and *kabuli* chickpea accessions were determined using the same set of SNPs analysed earlier. High natural allelic diversity based on a broader range of genetic distance was detected especially by MAD-SNPs that varied from 0.12 to 0.79, with an average of 0.67 (Fig. 3 and Table 2). All DAD-, KAD-, MAD- and TAD-SNPs exhibited a relatively wider range of allelic diversity in 189 *desi* chickpea accessions than that of 102 *kabuli* accessions. As compared to others, MAD-SNPs had maximum potential to detect a wider range of genetic distance and thus revealed high natural allelic diversity among accessions belonging to *desi* (mean genetic distance: 0.69) and *kabuli* (0.58) chickpea (Fig. 3 and Table 2). TAD-SNPs detected low allelic diversity which is reflected from narrow range of genetic distance among accessions within *desi* (mean genetic distance: 0.39) and *kabuli* (0.31) chickpea.

The population genetic structure among 291 accessions with the genotyping information of aforesaid SNPs classified all these accessions into two distinct population groups at a K (population number) value of 2 as POP and POP II (Fig. 3). The POP I included 73 *kabuli* and 29 *desi* accessions while POP II comprised of 129 *desi* and 60 *kabuli* accessions. The K value outcome with the best replicate was ascertained by estimating the average LnP(D) (log-likelihood) (exhibiting the greatest apparent inflection) and the second order statistics of ΔK (showing a sharp peak with maximum value of ΔK). All these SNPs detected a higher polymorphic potential in POP II (mean PIC: 0.31–0.43) than that of POP I. A significant population divergence based on pair-wise FST (P < 0.001) was estimated as 0.69 between POP I and POP II. Highest F_ST_-based population differentiation of 0.30 was observed within POP II. All 291 *desi* and *kabuli* chickpea accessions clearly belonged to a structured population in which 69% of their inferred ancestry was derived from one model-based population and the remaining 31% had an admixed ancestry (Fig. 3). Maximum admixed ancestry was observed in POP II (28%) and minimum in POP I (17%). Admixed ancestry was estimated as 39% between POP I and POP II. We could not detect admixture in 19.2% of chickpea accessions while 23% of accessions exhibited up to 20% admixed ancestry.

### Genome-wide association study (GWAS) of seed yield in chickpea

**Figure 4:**
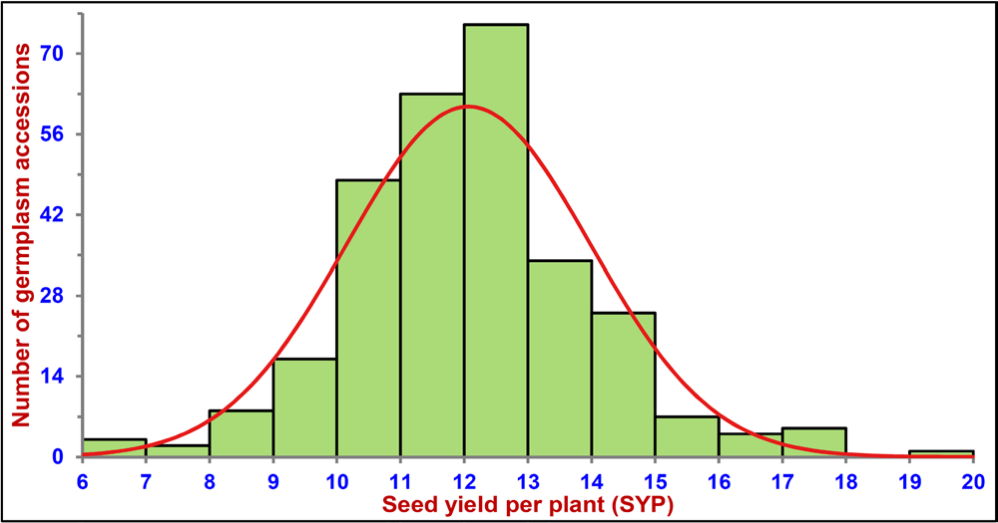
Frequency distribution of seed yield per plant (SYP) trait variation estimated among 291 *desi* and *kabuli* chickpea germplasm accessions (association panel) field phenotyped at multiple geographical locations/years.

Association mapping was performed by correlating the genotyping information of chromosome-wise physically mapped SNP-types (619 DAD-, 531 KAD-, 884 MAD- and 1083 TAD-SNPs) and replicated multi-location/years seed yield per plant (SYP) field phenotyping data of 291 *desi* and *kabuli* chickpea accessions (association panel). The use of these SNPs classified these *desi* and *kabuli* accessions into aforesaid two distinct population groups (POP I and POP II) (Fig. 3). A wider level of significant phenotypic variation for SYP trait varying from 6.2 to 19.3 g with the mean ± standard deviation of 12.1 ± 1.9 and coefficient of variation (CV) of 15.7% as well as high broad-sense heritability (H^2^: 86%) was observed among accessions belonging to the association panel based on multi-environments field data. A normal frequency distribution of quantitative SYP trait among accessions of an association panel was evident (Fig. 4). This altogether infers an existence of a broader natural phenotypic diversity level for SYP trait in these accessions representing diverse eco-geographical regions of the world. These screened germplasm accessions therefore, can be included in an association panel for quick mining of novel functional allelic variants to associate these markers most efficiently with SYP trait at a whole genome and/or gene level in chickpea.

**Figure 5:**
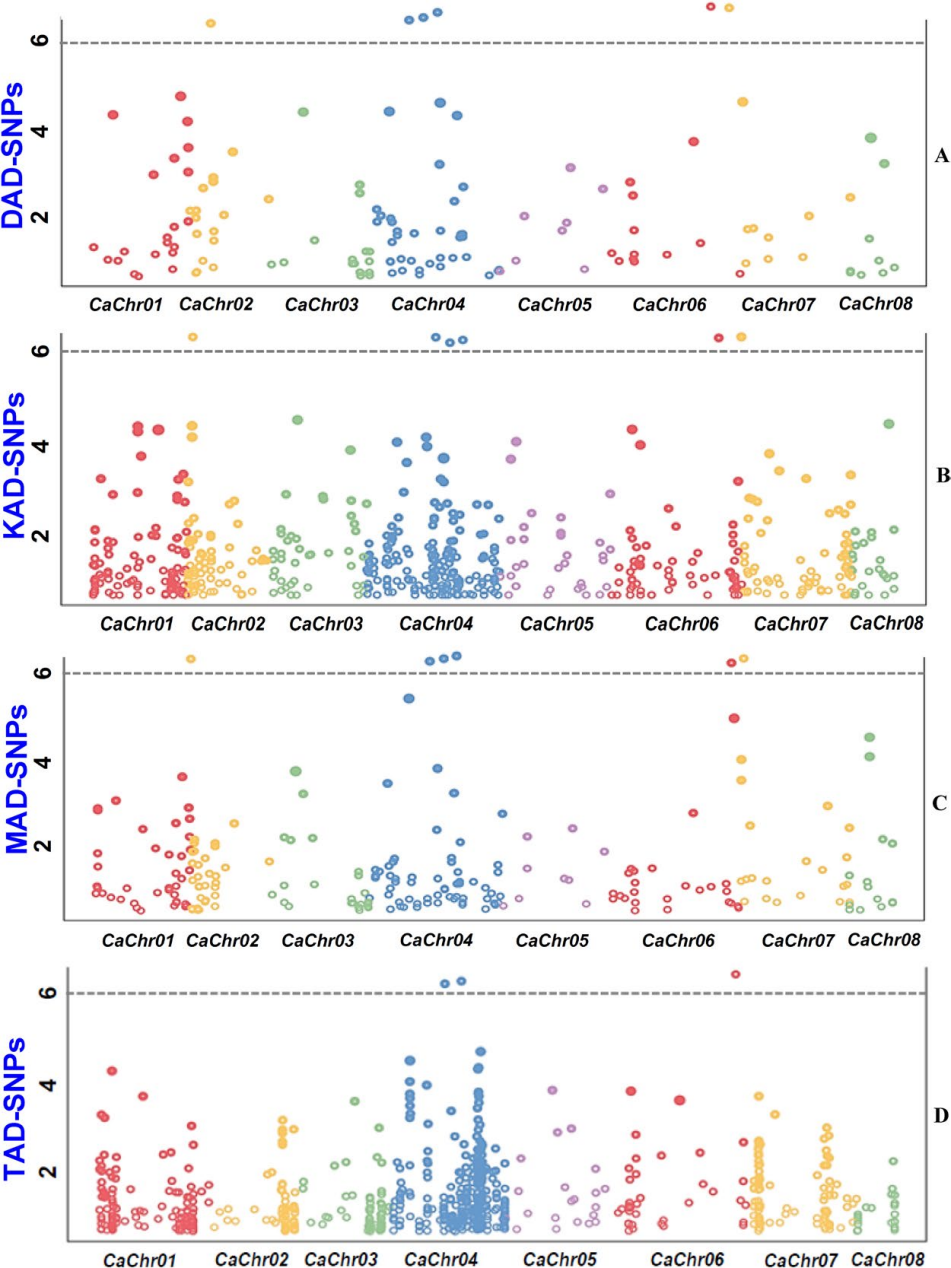
GWAS-derived Manhattan plot depicting significant P-values associated with seed yield per plant (SYP) using 619 DAD-, 531 KAD-, 884 MAD- and 1083 TAD-SNPs in chickpea. The genomic distribution of SNPs physically mapped on eight chromosomes of chickpea genome are designated by the x-axis. The y-axis specifies the −log_10_ (P)-value for significant association of SNP loci with SYP trait. The SNPs significantly associated with SYP trait at a cut-off P value ≤ 10^−6^ are highlighted with dotted lines.

The genetic association analysis at a false discovery rate (FDR) cut-off ≤0.05 using the genotyping data of 1967 SNPs identified six genomic loci exhibiting significant association with SYP trait at a P ≤ 10^−6^ (Fig. 5 and Table 3). Interestingly, the DAD-, KAD- and MAD-SNPs (six loci), as compared to TAD-SNPs (three loci), detected a higher number of significant SNP loci and therefore, these informative markers had a greater potential for SYP trait association in chickpea (Fig. 5). Considering a greater efficiency of MAD-SNPs in detecting high intra-specific polymorphism and natural allelic diversity among accessions belonging to an association panel, a strong potential of these marker types for precise trait association mapping is expected at a genome-wide scale. These six SYP trait-associated SNPs were physically mapped on four chromosomes (2, 4, 6 and 7) of *desi* chickpea genome (Fig. 5 and Table 3). A maximum of three SYP-associated genomic SNP loci were mapped on chromosome 4. The identification of more than one SYP trait-associated genomic loci reflects complex genetic architecture of the studied trait, which was further dissected in our study through high-resolution association mapping involving four various kinds of informative natural SNP allelic variants (DAD-, KAD-, MAD- and TAD-SNPs). The detailed structural annotation of six SYP-associated genomic loci revealed the presence of five SNPs in the coding (two synonymous and three non-synonymous SNPs) and one SNP in the non-coding (intronic) sequence components of six genes (Table 3). The SYP trait phenotypic variation explained (PVE) by six gene-derived maximum effect SNP loci varied from 11.2 to 24.2% R^2^ (P: 2.3 × 10^−6^ to 1.2 × 10^−8^) among *desi* and *kabuli* accessions belonging to an association panel. The combined SYP trait phenotypic variation explained by all significant six gene-based SNP loci was 27.5%. Notably, one synonymous SNP derived from a gene encoding pentatricopeptide repeat (PPR)-containing protein exhibited strong association (24.2% PVE and 1.2 × 10^−8^ P) with SYP trait in chickpea (Table 3). The outcome of aforesaid association study was further revalidated by a multi-locus association mapping which identified 6 quantitative trait nucleotides (QTNs) for SYP trait varying from 4.8 to 10.3 LOD (logarithm of odds) and 3.5 to 6.9% R^2^. To ascertain the trait association, SNPs identified in the 100 kb sequences flanking either side of these six SYP-associated QTNs were used for association mapping. None of the SNPs beside trait-associated SNPs was identified to be significantly associated with SYP trait in chickpea.

**Table 3:** Genomic SNP loci significantly associated with seed yield per plant in chickpea.

SNP IDs	SNPs	SNP physical positions (bp)	*Desi* chromosomes	Gene accession IDs	Structural annotation	Encoded amino acids	Putative functions	CMLM		FASTmrEMMA			
								P	PVE (R^2^%)	LOD	Effect	MAF	r^2^ (%)
MAD_SNP155	[G/A]	2285980	*Ca_Desi_Chr02*	Ca_03044	CDS (SYNONYMOUS)	—	Pentatricopeptide repeat (PPR)-containing protein	1.2 × 10^−8^	24.2	10.3	−0.310	0.24	6.9
MAD_SNP415	[A/G]	16444752	*Ca_Desi_Chr04*	Ca_09334	CDS (NON-SYNONYMOUS)	TGT (cysteine) - CGT (arginine)	Two-component response regulator (ARR2)	2.1 × 10^−7^	13.4	6.7	−0.253	0.27	4.3
MAD_SNP472	[G/C]	27958812	*Ca_Desi_Chr04*	Ca_10271	CDS (NON-SYNONYMOUS)	ACT (threonine) - AGT (serine)	Acyl-[acyl-carrier-protein] desaturase, chloroplastic	2.3 × 10^−7^	12.4	5.5	−0.217	0.20	3.9
MAD_SNP522	[A/T]	34596600	*Ca_Desi_Chr04*	Ca_10851	INTRON	—	Mediator transcription factor	1.5 × 10^−8^	20.1	8.7	−0.243	0.21	5.8
MAD_SNP746	[C/G]	52329312	*Ca_Desi_Chr06*	Ca_18831	CDS (SYNONYMOUS)	—	Arginine decarboxylase (ADC)	1.5 × 10^−7^	14.7	7.2	−0.231	0.23	5.2
MAD_SNP757	[G/C]	1103940	*Ca_Desi_Chr07*	Ca_19072	CDS (NON-SYNONYMOUS)	GAC (alanine) - GAG (glutamine)	Unknown expressed protein	1.4 × 10^−7^	11.2	4.8	−0.211	0.20	3.5

## Discussion

Genomics-assisted crop improvement of chickpea is usually hindered due to its narrow genetic base and low intra-specific polymorphism among cultivated *desi* and *kabuli* accessions. To overcome these, large-scale discovery and genotyping of informative sequence-based markers like SNPs differentiating the maximum number of *desi* and *kabuli* accessions together and exhibiting high intra-specific potential among cultivated accessions using a user-friendly, rapid and cost-effective genotyping assay is essential at a genome-wide scale^14,18,45^. This will essentially accelerate the generation of genotyping information of numerous genome-wide SNP markers differentiating large-scale accessions for high-resolution association mapping and efficient dissection of complex quantitative traits in order to drive genetic enhancement of chickpea. In this perspective, validation and genotyping of numerous non-erroneous and robust SNPs that are discovered by *desi* and *kabuli* cultivar-wise individual assembling of low-sequencing depth-coverage sequence reads generated from numerous germplasm accessions using GBS assay, can be an efficient alternative genome-wide strategy in chickpea. Adopting this strategy, the current study has developed diverse kinds of genome-wide 1967 SNPs including 884 DAD-, KAD-, MAD-SNPs and 1083 TAD-SNPs to evaluate their potential for discriminating cultivated *desi* and *kabuli* accessions and exhibiting association with SYP traits in chickpea. The structurally and functionally annotated informative natural SNP allelic variants discovered by us can be utilized for rapid identification of functionally relevant molecular tags associated with multiple agronomic traits in chickpea. These developed SNPs will further serve as a useful genomic resource for cultivar-wise selection of informative SNPs differentiating *desi* and *kabuli* accessions at a genome-wide scale to expedite high-throughput genetic analysis in chickpea.

The intra-specific polymorphic potential especially detected by SNPs among 291 cultivated *desi* and *kabuli* chickpea germplasm accessions was much higher as compared to that reported in earlier studies with SNP markers^3,45–48^. Nevertheless, the nucleotide diversity measured by DAD-, KAD- and MAD-SNPs among 189 *desi* and 102 *kabuli* accessions was almost comparable to that documented using the genome-wide resequencing- and GBS-derived SNP markers^3,45^. The level of molecular diversity detected especially by MAD-SNPs among 291 cultivated *desi* and *kabuli* chickpea accessions is much higher as compared to that documented earlier with SNP markers^3,45–48^. Distinct differentiation and clustering of 291 accessions into two major population groups are in accordance with their cultivar-specific origin, pedigree relationships and parentage that well-corresponded with previous studies of Upadhyaya *et al*.^49^ and Kujur *et al*.^45^.

The clustering of accessions into a specific population group was strongly influenced by geographical origin as well as adaptive environment rather than their known parentage and pedigree. The observed admixed population genetic structure inferred the occurrence of multiple domestication events along with strong evolutionary bottleneck among *desi* and *kabuli* accessions. The continuous crop genetic improvement breeding efforts with an aim to develop high-yielding stress tolerant *desi* and *kabuli* cultivars may influence their domestication pattern and genetic structure immensely therefore, detection of numerous admixture traces among these cultivated 291 *desi* and *kabuli* chickpea accessions is expected. The determination of high-resolution population genetic structure among accessions is vital to assess the accuracy (non-spurious) of robust SNPs associated with a particular agronomic trait in crop plants. In this context, high-resolution admixed population genetic structure can essentially be effective in detecting non-spurious genomic loci associated significantly with target traits in chickpea.

While trying to evaluate the potential of the developed markers in explaining molecular diversity and population genetic structure in chickpea it was observed that large-scale genome-wide MAD-SNPs discovered by especially comparing the low-sequencing depth-coverage sequence-reads of multiple accessions exhibited a higher potential of intra-specific polymorphism as well as a wider natural allelic diversity than that of two accessions (TAD-SNPs). Therefore, these informative MAD-SNPs with the requisite potential to establish precise phylogenetic relationships and determine admixed population genetic structure among 291 *desi* and *kabuli* chickpea accessions can be deployed in marker-aided introgression breeding for varietal improvement targeting multiple traits of agronomic importance in chickpea.

The current investigation developed numerous informative DAD-, KAD-, MAD- and TAD-SNPs differentiating 291 precisely field-phenotyped *desi* and *kabuli* chickpea accessions at a genome-wide scale to evaluate their potential for marker-trait association and identify potential genomic loci and natural allelic variants governing complex seed yield quantitative trait in chickpea. The high-resolution association study altogether detected six genomic SNP loci including QTNs significantly associated with SYP traits. None of these six major SYP trait-associated QTNs identified has been documented by earlier association and QTL mapping studies in chickpea. This infers the novelty of the detected gene-based natural SNP allelic variants in governing seed yield trait in chickpea. Essentially, the five protein-coding genes with synonymous and non-synonymous SNP loci associated with SYP trait delineated by high-resolution association mapping have functional relevance for quantitative dissection of complex seed yield traits in chickpea. These SYP trait-associated SNPs will be of immense use in establishing marker-trait linkages and identification of natural allelic variants in the genes regulating SYP trait, thereby can accelerate the marker-assisted selection to develop desirable cultivars with high seed yield.

The delineated genes encoding response regulator, arginine decarboxylase, Mediator transcription factor and PPR protein with synonymous and non-synonymous coding and intronic SNPs associated with SYP trait in chickpea are known to control gene expression dynamics and transcriptional regulatory pathways underlying growth, development and yield traits in multiple crop plants^50–56^. Essentially, the role of delineated most-promising SYP trait-associated PPR genes with synonymous SNPs in regulating fertility restoration, heterosis breeding and hybrid seed production is well-documented in crop plants including legumes^52,54,56,57^. Modulation of their gene expression can thereby enhance the overall seed yield. Henceforth, functionally relevant molecular signatures including candidate genes, transcription factors and natural SNP alleles delineated in the present study can essentially be deployed for genomics-assisted crop improvement to develop high seed-yielding cultivars of chickpea.

## Methods

### Chickpea genetic resources

A total of 291 phenotypically and genotypically diverse accessions (representing diverse eco-geographical locations of the world) belonging to *C*. *arietinum desi* (189 accessions) and *kabuli* (102) cultivars were selected from available chickpea reference core/mini-core germplasm collections^49^ as per Kujur *et al*.^10,45^ (Table S5). Of these, 92 including 39 *desi* and 53 *kabuli* accessions were chosen as a representative set for large-scale mining and genotyping of genome-wide SNPs employing GBS assay in chickpea (Table S6). The genomic DNA was isolated from the young leaves of aforesaid chickpea accessions using a DNeasy 96 Plant Kit (QIAGEN, CA, USA) as per the manufacturer’s instructions.

### Selection and annotation of genome-wide SNPs

The raw FASTQ sequence reads generated by sequencing of 92 *desi* and *kabuli* chickpea accessions using GBS assay were acquired from NCBI SRA database (Accession ID SRX845396) and de-multiplexed into individual sequences by using their unique barcodes in accordance with Kujur *et al*.^10,45^. The high-quality sequence reads of 92 accessions after filtering with NGS QC Toolkit^58^, were grouped into *desi* (39 accessions) and *kabuli* (53) cultivar-wise separately and further mapped individually on *desi* chickpea genome sequences^4^ using BWA^59^. The SNPs were mined by *desi* and *kabuli* cultivar-wise comparison of the mapped sequences among accessions using Samtools (http://www.htslib.org) and subsequently high-quality SNPs supported by at least 10 sequence reads (with SNP base quality ≥20 and ≤1% missing data) were screened following Srivastava *et al*.^30^. A set of chromosome-wise uniformly mapped high-quality SNPs exhibiting differentiation only between two *desi* (ICC 4958) and *kabuli* (CDC Frontier) cultivars (which draft genomes are sequenced) were screened for further analysis. The structural and functional annotation of SNPs in diverse coding (synonymous and non-synonymous substitutions) and non-coding (intron, upstream/downstream regulatory and intergenic regions) sequence components of genes and genomes (chromosomes/pseudomolecules and unanchored scaffolds) were performed as per the *desi* (CGAP v2.0^4^) genome annotation.

### Large-scale genotyping of SNPs

For genotyping of SNPs, *desi* accessions (39)-derived (DAD) SNPs, *kabuli* accessions (53)-derived (KAD) SNPs, multiple accessions (92 *desi* and *kabuli*)-derived (MAD) SNPs and two accessions (ICC 4958 and CDC Frontier)-derived (TAD) SNPs were genotyped in the genomic DNA of 291 chickpea accessions using Sequenom MALDI-TOF MassARRAY assay (http://www.sequenom.com). The SNP loci along with their flanking 100-bp sequences were curated and a 34-plex MassARRAY multiplex iPLEX assay including adapter-ligated pre-amplification and unextended/nucleotide extension PCR primers targeting each SNP were designed by MassARRAY Assay Design software v3.1 following the criteria of Saxena *et al*.^60,61^. The multiplex PCR amplification, incubation, primer extension, resin clean-up and mass spectrometry-based SNP genotyping were carried out as per the manufacturer’s instructions of Sequenom iPLEX Gold amplification kit. The SNP genotyping data detected in iPLEX spectrochip bio-arrays were analysed by a MassARRAY Typer 3.4. The genotyping information of SNPs based on allele-specific differences in mass between extension products was visualized and documented among 291 chickpea accessions following Saxena *et al*.^60,61^.

For validation of high-quality SNPs, primer-pairs were designed targeting 100-bp either side flanking sequences of 192 representative SNP loci and PCR amplified using the genomic DNA of 48 selected *desi* and *kabuli* chickpea accessions. The amplicons were sequenced and high-quality sequences were aligned and compared among accessions to detect SNPs following Kujur *et al*.^8^ and Saxena *et al*.^60^.

### Assessment of polymorphic potential

The genotyping data of DAD-, KAD-, MAD- and TAD-SNPs were analysed with PowerMarker v3.51^62^ to estimate the per cent polymorphism and PIC (polymorphism information content) among 291 *desi* and *kabuli* chickpea accessions. The SNP genotyping information was further analysed on a 100-kb non-overlapping sliding window approach of TASSEL v5.0^63^ (http://www.maizegenetics.net) to measure one of the major nucleotide diversity parameter-θπ among chickpea accessions as per Kujur *et al*.^45^ and Bajaj *et al*.^18^.

### Determination of molecular diversity and population genetic structure

The genotyping data of SNPs were used to assess the molecular diversity and for establishing phylogenetic relationships among 291 *desi* and *kabuli* chickpea accessions. The cluster analysis among accessions was carried out based on Nei’s genetic distance^64^ using the neighbour-joining (NJ) method (with 1000 bootstrap replicates) of PowerMarker, and unrooted phylogenetic tree was constructed employing MEGA v7.0^65^.

To determine the population structure among 291 chickpea accessions, the genotyping data of SNPs were analysed in a model-based program STRUCTURE v2.3.4^66^ as per Kujur *et al*.^45^. The optimum population number (K) was determined following the *ad hoc*^66^ and *delta* K^67^ methods. Using the optimum K, the population structure model representing better relationships among chickpea accessions was determined. Diverse population genetic parameters including genetic divergence (FST) and degree of admixture among different population groups were estimated.

### Phenotyping of a constituted association panel

For large-scale phenotyping, an association panel consisting of 291 diverse *desi* (189) and *kabuli* (102) germplasm accessions were planted in the experimental field with 35 × 10 cm row-to-row and plant-to-plant spacing, respectively, following standard agronomic practices. These accessions were grown in the field as per ‘*alpha-lattice’* design with two replications during crop season for two consecutive years at two diverse geographical locations (New Delhi; latitude/longitude: 28.4°N/77.1°E and Hyderabad; 17.1°N/78.9°E) of India. Four selected *desi* (ICCV 10 and ICCV 93954) and *kabuli* (ICC 12968 and Annigeri) chickpea accessions sown after every 10 individual plants were served as reference in the field experimental design to ascertain the homogeneity of association panel across two environments. Seed yield per plant (SYP) was estimated by measuring the average weight (g) of fully matured dried seeds (at 10% moisture content) harvested from 10–15 representative plants of each accession. The genetic inheritance pattern of SYP trait based on diverse statistical parameters including coefficient of variation (CV), broad-sense heritability (H^2^) and frequency distribution were estimated in an association panel as per Bajaj *et al*.^18^ and Das *et al*.^23^.

### Association analysis

For efficient trait association mapping, we employed the environment-wise SYP phenotyping data measured across two diverse geographical locations and experimental years from each of the selected accession for estimating the mean SYP trait value in an individual accession. The SNP genotyping data was correlated with multi- environments replicated field phenotyping data of SYP trait as well as population structure (Q), kinship (K) matrix and principal component analysis (PCA) (P) information of 291 *desi* and *kabuli* accessions belonging to an association panel of chickpea. To perform association analysis, the compressed mixed linear model (CMLM) (P + K, K and Q + K) and population parameters previously determined (P3D) interfaces of GAPIT were utilized as per Thudi *et al*.^14^, Kujur *et al*.^10^ and Kumar *et al*.^65^. To determine the accuracy of SNP marker-trait association, we performed the quantile-quantile (Q-Q) plot-based false discovery rate (FDR cut-off ≤0.05) corrections for multiple comparisons between observed/expected −log10(P)-values and adjusted P-value threshold of significance in accordance with Benjamini and Hochberg^68^. The SNP loci associated with SYP trait at a lowest FDR adjusted P-value (threshold P < 1 × 10^−6^) and highest R^2^ were identified to be highly significant in chickpea. Following FDR-controlling method of model with the SNPs and adjusted P-value, R^2^ representing the magnitude of SNP marker-SYP trait association was estimated. The trait association outcomes were revalidated through a fast multi-locus random-SNP-effect EMMA (FASTmrEMMA) model of GWAS as per Wen *et al*.^69^. The QTNs with LOD >3.0 and P-value < 0.005 were identified to be significantly associated with studied trait.

## Electronic supplementary material

Table S1

Table S2

Table S3

Table S4

Table S5

Table S6
